# The Brussels and California Effects? Circular Economy Policy Influence Across Borders

**DOI:** 10.1007/s43615-025-00516-4

**Published:** 2025-02-26

**Authors:** Nancy Bocken, Matthew Coffay, Carl Dalhammar

**Affiliations:** 1 Maastricht Sustainability Institute, School of Business and Economics, Maastricht University, Tapijn 11 Building D, P.O. Box 616, Maastricht, 6200 MD The Netherlands; 2 International Institute for Industrial Environmental Economics (IIIEE), Lund University, Tegnérplatsen 4, Lund, Sweden; 3 Centre for Sustainable Business, NHH Norwegian School of Economics, Helleveien 30, Bergen, 5045 Norway; 4 Business School, University of Stavanger, Kjell Arholms gate 41, Stavanger, 4021 Norway

**Keywords:** Environmental policy, Circular economy policy, Innovation, Circular economy, Brussels effect, California effect

## Abstract

The influence of environmental policy has been known to move beyond the country or regional contexts in which they have been implemented. Examples in literature include the “California effect” and the “Brussels effect”, showing how the policies adopted in the EU and California influence other jurisdictions. In this paper we study the following: To what extent are companies operating in the United States influenced by circular economy policies outside their direct context? By interviewing companies operating in the United States, we find that firms are influenced by and actively work to influence circular economy policy, both as it originates from outside the United States (the Brussels effect, referring to policies in Europe) and from within the United States (such as the California effect). Key barriers to circular innovation include the lack of a comprehensive policy framework in the U.S., opposition from competitors, and making the business model work in the U.S. legal context. Strategies to overcome these include: getting legal support for circular business models, developing U.S. regulations, level the playing field for all U.S.-based companies, lobbying for supporting regulation, and collaboration. We find that EV battery recycling is a positive exception where U.S. policy provides clarity for circular innovation. Finally, we find that the characteristics of the ‘typical’ U.S. consumer may call for specific circular business models. We suggest future research to enhance our understanding on how policy might positively drive circular economy innovations in international companies.

## Introduction

In what we may label the “birth” of modern environmental policy in the 1960s and 1970s, the United States (U.S.) was considered the “green leader” [1]. The European Union (EU) had very few environmental laws adopted at that time. However, by the mid 1990s, the EU had started to adopt policies in many areas that were more progressive than its U.S. counterparts, whereas there was clear evidence that the pace of U.S. policymaking was stalling [1]. This development has continued, and today the EU can be considered as “the green leader” in most areas of policymaking to protect the environment and consumers [2, 3]. This includes the area of Circular Economy (CE), which has gained significant traction in European policy [4] and business [5]. There are several explanations for why EU policies are progressive [6]. First, the world’s “greenest” countries are EU members [7] which creates a fertile ground for adopting environmental policies. Second, and perhaps more importantly, national policies may pose a threat to the EU Internal Market, forcing producers to adjust to different obligations in the EU member states [8, 9]. This creates momentum for setting EU-wide policies to remove restrictions to trade. Recent examples include the mandatory French Repair Index and German obligations for companies to report on destruction of unsold goods. These national rules create problems for companies operating in multiple EU countries and have led to proposals for EU-wide laws [10]. Thus, policies in EU member states, and especially those with large markets, can trigger EU rules and policies to harmonize national policies that distort trade.

Importantly, Bradford (2020) claims that EU laws and policies are very influential *outside* the EU [3]. Companies adhere to EU standards to ensure that their products and services can be exported to the EU, and policymakers in several jurisdictions use EU policies as a ‘blueprint’ for domestic policies. Bradford (2020) labels this process “the Brussels Effect”, and the effect is most notable for regulations on products and chemicals, and related standards [3]. However, the Brussels Effect suggests that the policy context of influence surpasses national and state boundaries, and with global climate policies and global markets for goods and services, the influence of policy is growing and may come from unexpected angles. For instance, the Corporate Sustainability Reporting Directive (CSRD) of the EU [11], requiring detailed reporting of social and environmental issues related to goods and services offered by companies, also applies to imported goods and services. Indeed, it is estimated that the CSRD will force at least 10,000 companies outside the EU — of which a third are based in the United States — to issue independently verified sustainability disclosures [12].

This study seeks to explore to what extent the United States, both one of the largest economies and largest polluters [13, 14], is influenced by EU CE policy. Relevant CE policies include product regulation that supports durable, repairable and recyclable products, rules related to waste collection and recycling, public procurement that supports circular solutions, and taxes on waste and materials [15, 16]. While in Europe, the policy framework for the CE for companies in different sectors is becoming clearer, in the United States it is less clear [17].

It is easy to discount the importance of EU CE policy. However, here we want to investigate the role of CE policy transfer and the outsized influence that European CE policy might have on these larger economies (the Brussels effect), as well as how CE policy can emerge and spread within the United States. We investigate the following question: *To what extent are companies operating in the U.S. influenced by circular economy policies outside their direct context?*

This study seeks to contribute to a better understanding of the broader effects of circular economy policy for business, zooming into the United States as the world’s largest economy, but where CE policy is still largely absent. Section 2 provides the background, outlining the policy context in the EU and the United States. Section 3 outlines the qualitative research method. Section 4 presents the main results, while Section 5 provides a discussion, including implications for business and policy and suggestions for future research. Section 6 offers a conclusion.

## Background

### Circular Business Innovations & Policy

Circular economy as a concept has been spreading globally in different policy contexts [17, 18]. Europe, which benefits from the CE policy package as part of the Green Deal, has been seen as one of the circular entrepreneurial hotspots with many emerging circular start-ups [19]. At the same time, spurred also by organizations like the Ellen MacArthur Foundation, multinationals with European headquarters have started innovating for the circular economy and experimenting with circular business models [20]. One example of a platform enabling businesses in their transition is PACE, the Platform for Accelerating the Circular Economy, a public-private collaboration platform aimed at accelerating the circular economy transition [21].

In the United States, the CE is not embedded comprehensively in policy frameworks but in disparate policies related to waste or renewable energy provision. Yet, practices such as recycling and remanufacturing are well-established in industry, but often driven by economic motives in the absence of policies [22–24]. Indeed, some of the largest companies on the U.S. stock exchange have started innovating for the circular economy, but many of these innovations have focused on the lower-level R-strategies such as recycling and on efficiencies which cut costs at the same time [18, 25]. However, the more radical strategies around slowing the loop and sufficiency are still underexplored [18] with some exceptions, such as the outdoor company Patagonia’s comprehensive circular model including recycling, reuse and regeneration strategies [26]. Circular business model strategies are also beginning to emerge in the EV industry, with an increased focus on EV battery recycling due to IRA incentives [27]. Innovative companies are also emerging in this space, including Redwood Materials, which was founded by Tesla’s former CTO and has raised $2 billion in capital to date to develop battery recycling and resale capability for the first time in the U.S [28]. There is however some interest in promoting the CE at the federal level in the US, and research funding organizations have started to integrate CE challenges and topics in their practices [29].

How companies are influenced (indirectly) by circular economy policy frameworks to innovate their business models in terms of the value proposition (what product/service is offered to whom? ), value creation and delivery (how is this value provided? ), and value capture (how does the company make money and capture other forms of value? ) [30] is still poorly understood. Recent research based on a historical analysis of multinational corporations suggests that they may have the bargaining power to influence policy in the countries in which they operate [31]. However, this bargaining power depends on the institutional context in which they operate (e.g. nationalist economic policies may be unfavorable for multinationals’ bargaining power) [31]. This suggests that future research into the indirect policy influence on circular business model innovations might be a relevant research gap to explore. This may be of interest because of the strong policy drive for a CE in Europe.

Research shows that European companies, especially SMEs, with circular business models (CBMs) often need the support of public policies to be viable, or to scale up CBMs quickly [16, 20]. Examples include public procurement of remanufactured furniture, computers and tablets. There are public policies that support most CBMs — including product regulation that increases product quality and repairability, high level political targets for the circular economy, and public procurement processes that support CBMs — but there can also be public policies that support specific CBMs. The latter can include governmental support for specific policies like material passports and pre-audits for selective demolition of buildings to support re-use and recycling in the building sector, or government initiatives to support education and skills development in certain sectors [16]. Currently, the number of studies on the interaction between government policies and CBMs are limited, and only capture certain policies, and certain elements of companies’ business models [32]. Given that both business models and the policy framework surrounding businesses are dynamic and constantly evolving, it can be complex to study interactions.

### The CE Policy Context in the EU and United States

There are striking contrasts between CE policies in the EU and the United States. The EU embraced the CE concept early, whereas the CE concept has been catching on very slowly in the USA [17, 33, 34], though recently it has received more attention. The EU has adopted two CE action plans, containing proposals for ambitious legislative agendas. Furthermore, these CE action plans have been embedded in a comprehensive framework of policies to promote resource efficiency and address waste management [35]. These EU strategies have also resulted in several progressive policies, including right-to-repair (R2R) policies [36], and a new Battery Regulation that will enforce battery repairability for consumer electronics and mandate the inclusion of recycled content in large batteries. The EU has also adopted several ambitious legislative proposals, including a new Ecodesign Regulation that will be applied to set requirements on product lifetime and repairability for different product groups [37] as well as proposed a Green Claims Directive that aims to ensure the credibility of green claims and eco-labels [38]. EU member states have also adopted several progressive national policies [37, 39]. Thus, the EU — and its member states — have adopted several policies to move from the lower “R-strategies” (recover, recycle) to the higher ones (e.g. repair and re-use, and longer lifetimes).

In the United States, we see some related policy developments but mainly legislation regarding the right to repair, with developments at both the federal and state levels [36]. The EU has a more elaborate policy framework established for the CE than the United States and is more comprehensive in addressing issues related to products and waste management. It aims to advance the CE by regulating more aspects of products to support longer lifetimes, whereas the United States has mainly started to address right-to-repair (R2R) issues.

Table 1 exemplifies some of the differences between the EU and the United States and provides examples of relevant policies in California, which is a progressive state in terms of environmental regulation. If you compare EU member states to the U.S. states, EU member states appear to have developed more progressive CE policies. As an example, France has been a pioneer in establishing a mandatory Repair Index for products, and repair funds under its producer responsibility schemes [39], whereas Austria has introduced repair vouchers for electrical appliances [40], and France, Germany and Belgium have introduced policies to combat destruction of unsold/returned goods [41]. While several U.S. states have established product responsibility programs, most of them have proposed R2R bills, and some (including California) have addressed chemicals in products, but there do not seem to be any policies mirroring the latest developments among EU member states.

**Table 1:** CE elements in policy in Europe, the United States and California

CE element	Europe	European member states (sample)	USA	California
Examples of comprehensive policy frameworks and bills	Green Deal - Circular Economy Action Plan Indicators for the CE used to track CE progress Significant research funding for CE-related projects	Over 20 European countries have national roadmaps/ strategies, with slightly different foci, and action points e g Finnish roadmap to a circular economy 2016–2025– ‘Leading the cycle’– the first national roadmap French Roadmap for the Circular Economy adopted 2018 (Feuille de route pour l’économie circulaire)	CE initiatives are emerging but ad-hoc, no overall strategy Several high-level events to introduce the CE concept in the US Some US funding organizations are funding CE-related research IRA starts to address batteries in more comprehensive way	SB-707, Responsible Textile Recovery Act: apparel companies must establish collect/repair/recycle programs for old clothing and textiles. By 2026: brands must join or form a producer responsibility organization (PRO) to build the infrastructure for these initiatives By 2030: full implementation required, including drop-off and mail-in systems for consumers CalRecycle and other CA actors are increasingly referring to the CE in policy documents and speeches, though the focus of policies is mainly on waste and recycling, not on product law and product lifetimes.
Examples of frameworks for waste and recycling	EU producer responsibility laws for several product groups (including WEEE, vehicles, packaging, batteries, fishing gear, most recently textiles) ‘Recycled content’ obligations set for some batteries and PET bottles	France’s Anti-waste and Circular Economy Law is unique and has helped France being the first country adopting certain policies, e g banning planned obsolescence, banning destruction of unsold goods, adopting a mandatory repair index, and adopting strict rules on packaging. Several EU countries have adopted national producer responsibility laws for additional product groups Several EU countries, regions, and cities have established other policies that supports re-use and repair	The Resource Conservation and Recovery Act (RCRA) covers certain issues (e g toxic waste), but no national law on recycling US EPA has issued progress report on building the CE (2022) US EPA has issued recycling strategy part I, related to the CE (2021) The US EPA has announced that a draft national strategy for electronics will be published soon Producer responsibility laws are set by the States, with diverging practices throughout the US IRA has comprehensive policies for supporting battery recycling	California has introduced producer responsibility schemes for 6 product groups including packaging
Examples of policy frameworks for addressing plastic pollution	Plastics Strategy The Single Use Plastics Directive bans certain plastics, mandates recycled content in some packaging, and sets high recycling obligations	France has set the objective of ending all single-use plastic packaging on the French market by 2040. In order to achieve this objective, France set reduction, reuse and recycling targets for 2021–2025. There are also bans on certain plastic products, including microplastics, and recycling obligations. Several EU countries have adopted national strategies/roadmaps for plastics. Several European countries have national rules on micro plastics, plastic bags Several European countries have adopted taxes on plastic bags	Executive order NO. 3407 outlines “soft approaches” towards addressing the plastic issues, including using public procurement and better recycling infrastructure. The US EPA’s National Strategy to Prevent Plastic Pollution	Senate Bill (SB) 54 is considered a game-changer, as it introduces stringent rules for especially plastic packaging, as it sets stringent recycling targets and requires packaging to be compostable or recyclable. It is complemented by e g Bill 1383 on diverting some waste from landfills. Senate Bill 270 bans single-use carryout bags for certain businesses, instead requiring stores to sell reusable plastic bags made with recycled content or recycled paper bags. CA was the 1st US state to introduce a ban on certain plastic bags.
Reporting	CSRD of which’ “ESRS E5 Resource use and circular economy” is a part Various rules on conflict minerals, timber, import of products made with forced labour and products causing deforestation	The French Code of Commercial Law include specific requirements for a circular economy chapter in corporate sustainability reports. This chapter needs to include information about a qualifying company’s activities in the areas of waste prevention and sustainable resource use.	Federal rules on reporting about timber and conflict minerals	N/ A. The California Transparency in Supply Chains Act (Supply Chains Act) mainly relates to trafficking and slavery. It applies to large businesses that do business in CA.
Right-to-repair (R2R) policies	The EU is supporting R2R throughs several policies, such as the Ecodesign Directive (mandates e g provision of spare parts and software updates); mandatory labeling (e g, new energy label for smartphones will have a repairability score); consumer law (improved guarantees and repair options)	European countries have implemented several national policies, including: A mandatory repair index (FR) Obligatory guarantees on some repaired and secondhand products (FR) Repair vouchers (AUS) Reduced value added tax for repairs (SWE)	President Biden’s executive order to the Federal Trade Commission (FTC) to draft “right-to-repair” rules (2021) FTC policy initiatives and lawsuits to combat repair restrictions	Senate Bill No. 244 introduces R2R law in CA from 2024, to make it easier for owners to repair devices themselves or take them to independent repair shops. This Bill is expected to have a large influence in other US states
Product regulation on ecodesign	Mandatory energy efficiency standards are set for several product groups Mandatory energy labeling applies to several product groups The EU has set ecodesign criteria for lifetime and repairability for some product groups The new Ecodesign for Sustainable Product Regulation can be used to set ecodesign standards for almost all kinds of products	European countries are not supposed to adopt regulations that act as barriers for free movement within the EU, but has still adopted some relevant laws. Criminalization of planned obsolescence (FR) Longer mandatory consumer guarantees (3–5 years, adopted by several countries)	Mandatory energy efficiency standards are set for several product groups Mandatory energy labeling applies to several product groups Ecodesign obligations only relate to minimum energy efficiency standards, for energy using appliances	Rules on State Regulated LED Lamps (SLED) in California require a minimum lifetime of 10,000 h Consumer rights, and guarantees, vary between US states

Note. The table is based on online information at the web pages of: the state of California, CalRecycle, the USEPA, the European Commission, the Ellen MacArthur Foundation, the US government, and the White House. In addition, it is based on: [16, 36, 42, 43]

### Gap: Learning Across the United States and Europe

From the above it is clear that the CE policy framework is more comprehensive than in the United States but the fact that the latter is a laggard needs some qualification. Several U.S. policies have begun to emerge which result in CE outcomes, but which are not broadly marketed or seen as ‘CE policies’ per se. For example, the Inflation Reduction Act (IRA) (largely seen as climate-focused, as opposed to e.g. the EU Circular Economy Action Plan with a clear CE focus) contains a provision which incentivizes U.S. electric vehicle (EV) manufacturers to use reclaimed battery materials from decommissioned batteries [27, 44]. EV manufacturers utilizing recycled materials can claim them as U.S. origin (even if they originated outside the U.S.), which in turn allows those manufacturers to claim valuable subsidies under the IRA. This has resulted in a rush to construct battery recycling facilities, possibly putting the United States ahead of Europe in long term battery recycling capacity [45–47].

Thus, U.S. environmental leadership is still possible. Furthermore, some U.S. states, most notably California, have adopted a range of progressive environmental policies. State policies, especially those adopted in large markets like California, often have implications in other parts of the United States and provide a blueprint for other states’ policies, a process often referred to as the “*California Effect”* (somewhat similar to the Brussels Effect, but in the US context) [48, 49]. The work by Vogel (1997) even illustrates how California’s strict automotive emissions standards influenced other countries, like Germany (and later the EU), Japan and South Korea, which ended up with similar standards [49, 50]. The size of California’s economy is a determining factor for why it can affect actors outside its borders. Were California a country, its GDP of over $3 trillion would make it the world’s fifth largest economy [51].

Yet, in comparison to the EU, the United States has adopted few CE policies at the federal level (where policies mainly address waste management) and limited policies among the states. U.S. interest for the CE is increasing at the highest political level, and among research funders [29], but this is not yet matched by national policies similar to those in the EU. Regarding producer responsibility rules — mandating producers to collect and recycle used packaging and other products — there have been problems in adopting federal legislation in both the United States and Canada, but some states and provinces have adopted their own laws [43]. Also, for chemicals in products, the rules differ throughout North America [43].

Researchers have identified both a California effect [48–50] and a Brussels effect [3] in terms of environmental policy spillover. While the former is much older, the latter is newer and could be highly relevant in the circular economy context, allowing policies to have impacts in jurisdictions where domestic policies are lacking. At the same time, we see that California has also modeled their policies on EU policies. As an example, recent California bans of certain additives in cosmetics have followed the EU regulation, while Maryland is adopting similar rules as California [42]. In the EU, there is some cross-country policy learning and diffusion as well. For instance, France has a Repair Index, and now other countries like Spain are planning to introduce similar policies [37]. However, EU industries are worried about these national policies, as they can be costly for companies having to comply with national laws on products and/or undertake costly measures to provide mandatory information about their product in different national markets, and thus we see them lobbying for EU-wide rules, or that the EU should not allow certain national schemes that are undermining free movement of products [37]. We also see how the European Commission has addressed this through proposing several EU policies that can harmonize national rules [37].

Bradford [3] shows how the Brussels Effect works through two distinct processes: Companies outside the EU can voluntarily adopt practices that adhere to EU rules (e.g. removing substance from a product that is banned in the EU), as they want to be sure to be able to sell their products in the EU; this is referred to as a “de facto” Brussels Effect. But EU laws and policies can also spread to other jurisdictions if non-EU countries adopt laws that are similar in nature to those of the EU; a “de jure” Brussels Effect. They may do so for at least two reasons: (1) the EU is seen as a “green leader”, generally, and thus it makes sense to mimic its policies; (2) mimicking EU policies supports domestic companies’ potential to export to the EU. The two mechanisms work in synergy. For instance, a non-EU company that has chosen to adjust their practices in accordance with EU law requirements have reasons to lobby for national rules that are similar to EU rules, as this would require their domestic competitors to comply with more demanding rules.

Importantly, Bradford [3] identifies five elements which underlie the existence of the Brussels Effect: market size; regulatory capacity; stringent standards; inelastic targets; and non-divisibility. Market size is a straightforward and easy to understand explanatory element. However, taken on its own, market size is a necessary but insufficient condition. For the Brussels Effect to occur, the other elements must also be present. Regulatory capacity refers to the ability of a jurisdiction to create and enforce regulations [3]. Stringent standards are related to but distinct from regulatory capacity, in that they refer to a jurisdiction’s ability to not only create regulations, but to harness the necessary political will to enforce them [3]. Inelastic targets are those targets which a given regulation aims to impact: capital is elastic insofar as it can flee to another jurisdiction to avoid regulation, while consumers are inelastic as they cannot simply move their consumption to another jurisdiction [3]. Non-divisibility is the notion that a multinational corporation opts to apply a given regulatory standard not only to its activities in Europe, but to its global operations [3], typically because the benefits of adhering to a single standard outweigh those of adopting one standard for e.g., the EU market while adopting a more lax standard for other markets.

The research on the Brussels and California Effects are part of the larger scholarship related to theories on ‘policy transfer’ [52] or ‘policy diffusion’ [53]. There is much confusion and overlaps related to this terminology in the literature. What we mean with policy diffusion in this paper is the phenomena where one jurisdiction adopts similar policies, laws, or policy interventions (i.e. policy instruments, such as a tax or an eco-label) as those already adopted in another jurisdiction. This diffusion (or transfer) can be a copy of the existing policy or, more commonly, adopted with some variations to better fit the domestic context. Diffusion can happen between countries, but also between other jurisdictions (e.g. California adopting similar policies as the EU ones), or even between cities. There are two main drivers for diffusion of environmental policies [53]: regulatory competition and ideational competition. Regulatory competition is strongly related to issues like global trade and economic competition and explains a lot of the allure of EU and California policies: other jurisdictions adopt them in order to improve market access and increase competitiveness of domestic firms. Ideational competition is a process where certain policies are attractive as they reflect “best practice” and are seen as expressions of good policy ideas. When California adopts similar policies as those found in the EU, it is probably in many cases because they consider EU policies to be best practice and because the EU and California have similar visions of sustainability [54].

In order for policies to diffuse, there is a need to have “green leaders”, or “green pioneers”, that are first adopters of new policies and laws [53, 55]. In the EU, France currently acts as a leader in setting new CE policies and laws [56]. In this way, France pushes the EU to adopt more stringent policies, but this also leads to conflicts as it can distort trade within the EU [57].

While the policy context is complex, with a global economy and internationally operating companies, it is important to understand the (in)direct effects of environmental policies. Here we focus on emerging European circular economy policy and how it might affect companies in the United States operating in a more fragmented local circular economy context.

This paper studies to what extent companies operating in the United States are influenced by CE policies outside their direct context. We also reflect on the existence of the five elements by Bradford [3] underscoring the Brussels effect: market size, regulatory capacity, stringent standards, inelastic targets, and non-divisibility.

## Method

Not much research (if any) has been done on how companies in the United States have been influenced by CE policy in Europe. Hence, this research is of an exploratory nature to understand to what extent U.S. companies are influenced by circular economy policies inside as well as outside their direct context. We adopted a qualitative approach to the research, opting for an open-ended and exploratory approach to conducting semi-structured interviews. Broadly, qualitative approaches to research in the social sciences have been recognized for their ability to help address Grand Societal Challenges [58]. As contrasted with traditional approaches to building theoretical and empirical insight, exploratory qualitative approaches allow for more diverse ways of engaging in knowledge-building, going beyond the predictive promise of theory-building to challenge conventional understanding and foster change [59].

We asked CE innovators in U.S.-based companies to what extent they influence and are influenced by their institutional context, including policies and regulations. We selected companies that had successfully implemented CE innovations in the US context, so they could express their experiences with what barriers they might have experienced while implementing CE innovations, the ways in which they had overcome these barriers, and the role of (CE) policy. We also looked for a heterogeneous example to cover a breadth of sectors and organization types and get a broad picture of the influence of CE policy influence. Table 2 includes the interviewee sample: the industry, type of organization, the circular business models discussed during the interview, and the place of the head office.

**Table 2:** Interviews

Interview	Industry	Size/ type	Circular business models discussed	Head office
A	Furniture	Multinational	Secondhand model	Europe
B	Furniture	Large international business	Product longevity & premium pricing	UK
C	Healthcare	Multinational	Service model, take-back model	Europe
D	Consumer and Business furniture	Scale-up business	Service/ rental model incl. Repair & refurbishment	USA
E	Office furniture	Large US business	Take-back, Remanufacture & Refurbish model of other OEMs’ products	USA
F	Office supply retail	Large international business	Platform enabling remanufacturing of other OEMs’ products	USA
G	Engineering equipment	Multinational business	Remanufacturing model (of own products)	USA
H	Electronics recycling services	Large US business	Recycling and remanufacturing other OEMs’ products	USA
I	Environmental assessment services	Startup company	Supporting assessment of impact of CBMs	USA
J	Document and printing services	Multinational business	Remanufacturing model (of own products)	Asia
K	Fashion	Multinational business	Product longevity & premium pricing	Europe
L	ICT services	Multinational business	Enabling CBMs through software solutions and consultancy	USA
M	Mobility	Multinational business	Remanufacturing model (of own products)	Asia
N	Reuse ecosystem for consumer products	Scale-up company	Platforms enabling reuse and recycling	USA
O	Furniture Rental	Scale-up company	Service/ rental model incl. Repair & refurbishment	USA
P	Consumer electronics	Large international company	Remanufacturing model (of own products)	UK

Companies were contacted from the first author’s network to identify those companies who pursue CE innovations in the United States. Some snowballing took place to expand the sample and identify successful CE examples not yet familiar to the authors [60]. The questions were exploratory in nature that allows for “opportunities for a narrative to unfold” [61, p. 2] and revolved around:

- The drivers and barriers to CE innovations.
- The role of policy related to their circular economy innovations.
- The extent to which their company was involved in lobbying to shape policies and regulations, or to receive preferential treatment, specific subsidies, and rewards [62, 63].

Interviews took on average 54 min. Data were independently coded by the first and second author, and subsequently discussed by all authors for reliability purposes. The authors coded the data for the following themes [64]: evidence of the Brussels effect, the California effect, and barriers and strategies to overcome these, related to legislative aspects. Moreover, as appropriate in qualitative data analysis, notes were taken on emerging themes originating from the interviews.

## Results

The interviews revealed a number of key insights related to how U.S. companies are influenced by and actively work to influence circular economy policy, both as it originates from outside the United States (e.g., the Brussels effect) and from within the United States (e.g., the California effect). Interviewee responses conformed to a number of themes, including legislative barriers (Table 3); strategies to overcome these barriers (Table 4); the Brussels effect (Table 5); the California effect (Table 6); the special case of EV battery recycling, and consumer demand. Representative quotes from the interviews are represented in Tables 3, 4, 5 and 6.

**Table 3:** Legislative barriers

Barriers	Illustrative interview quotes
Lack of comprehensive policy framework in the US	*“And where the US needs to be better about what we’re doing because we’re not having the infrastructure. Certainly not the infrastructure that Europe has*,* nor the policy*,* nor the legislation which is advancing. What’s going on there. We need to find alternatives to make it better. That’s really where a lot of our discussions are.”* (interview A) *“I don’t think we’ve gotten that far in the US. I mean*,* sometimes we get The State of California has some pretty strict guidelines around what they can and can’t use*,* so we will have to sometimes provide them with like*,* oh*,* this this will last for this long*,* and this is non toxic. (…) But it’s not something that comes up very frequently here in the US”* (Interview B).
Making the business model legally work in a US legislative context	*“There are some languages in some States that prohibit some buyback resale structures based on the other types of buy back resell structures. So we’ve worked with [regulatory agencies] for them to understand that we’re only buying back our own product.”* (interview A) *“we’ve spoken with some legislators in the past about this and and you know*,* that is*,* that’s always the the big debate*,* you know*,* with like innovative business models and technology*,* and just like regulation and legislation* (interview D)
Opposition from other industry actors	*“So our sector was not embraced by the OEMs. They constantly tried to throw obstacles in our way. Performance*,* optical*,* safety issues where we constantly had to prove we want the same level playing field”* (interview E). *“But then*,* when there was*,* there was pending legislation*,* you know*,* and whether it was at the State level or the Federal level*,* they [competitors] would say*,* Look*,* there’s no need to regulate this. Look what [Company F] is doing. They’re doing this voluntarily. Right?”* (Interview F)

**Table 4:** Strategies to overcome barriers

Strategies to overcome barriers	Illustrative interview quotes
Get legal support for a business model	*“So what we’ve had to do in some States is work with policy and policymakers and regulatory agencies to get the legal support to be able to do this. There are some languages in some States that prohibit some buyback resale structures based on the other types of buy back resell structures. So we’ve worked with them*,* for them to understand that we’re only buying back our own product […] So we did have to change legislation*,* which is quite a bit disruptive…* (interview A) *“innovative business models in the United States*,* [are] a little bit trickier to navigate like…. You need to know when the furniture was produced. What year*,* when*,* how many people have utilized it*,* how many turns it’s had*,* and that needs to be disclosed*,* all that makes sense in theory. But when it comes to antiques*,* we maybe will find a dealer that knows the exact year that something was maybe built.* (interview D)
Developing own rules and regulations	*“Where we actually brought government agencies some academia and private sector folks together under […] leadership*,* and we had another general meeting*,* and we launched Remanufacturing Industry Council 3.O”* (interview E) *“We all need to stay united on this so that we don’t face these other pieces of legislation now in Europe. Obviously you had REACH*,* and there was. You know*,* you’ve got the WEEE directive. But in the United States there was really none of that. And so my point was*,* look*,* I think we should create a voluntary program if we want. And people that want to […] make that business work”.* (interview F)
Emerging legislation / leveling the playing field	*“So if you go to [industry association website] look up their sustainability standard level you’ll see that our name is listed there for a number of product lines*,* and that basically gets us on the same playing field as the OE[M]s and presenting to clients and solutions”* (interview E). *“The United States has a large focus [is] on low and embodied carbon materials in general. So the US Government has actually put*,* you know*,* requirements out to look at low embodied*,* you know*,* carbon alternatives. […] So I think. I think this whole focus on scope 3 by companies and governments […] is going to drive a lot of circularity”.* (interview F)
Collaboration	*“We did a lot of work with [NGOs and other institutes] for example*,* and go globally with folk*,* you know*,* with companies like [X and Y]*,* you know*,* to make sure that that we weren’t creating opportunities for illegal timber to get into our supply chains and created*,* you know*,* transparency working through global forest and a range of other programs. But I found that those were you know those kinds of collaboratives were really important in terms of setting the setting the standards by which the marketplace in the future would be built right and also create started to create transparency and multi-tier supply chains that we’re lacking up until that point* (interview F)
Supporting regulation	*“We have […] dealers all over the world*,* so we’re trying to make it as convenient as possible for our customers*,* because the more convenient*,* the more likely they are going to be able to bring that core product back..So if you have regulations in those countries that support the free flow of the core [to be remanufactured automotive part]*,* then that really creates that whole circular economy for us….*,* where there are no restrictions on remanufacturing or the remanufacturing business model*,* then those countries and those citizens of those countries really help benefit that circular economy because they’re able to. Those products can perform*,* you know*,* like new*,* like*,* I said*,* at a lower price”.* (interview G) *“Yeah*,* I would just harp on the fact that if it is legally mandated…companies will do it. So the challenge is getting legislation to kind of catch up with where the rest of the world is…There’s just a lot of challenges being a country made up of a lot of states that have their own regulations and standards. That we kind of have to figure out.”* (Interview P)

**Table 5:** Brussels effect

Finding	Illustrative interview quotes	Five elements of Bradford [3]
European environmental legislation	*I think [legislation] starts in Europe*,* and they will make their way to the US. So I think definitely legislation*,* I think I see California as sort of a mirror of Europe a little bit… So yeah*,* I think legislation and stuff like that can really drive*,* drive*,* change* (Interview C). *I feel strongly influenced over here. I think we people in this movement*,* you know*,* and sustainability in the US. Are influenced and need to be aware of the activities in Europe. CSRD..* (Interview I) *“We do believe that the EU is leading the world in this with these directives. If US brands wanna do business in Europe*,* they’re gonna have to learn the ways of the Europeans.”* (Interview N)	*Market size* *Regulatory capacity* *Stringent standards*
Trickle down from European headquarters	*“So it is starting to live at the leadership level […] In the last year*,* of the 6 factories left*,* 2 were in Europe because they had a very complex type […] of waste*,* but the rest was in the US. And that was because a lot is so cheap there. And a lack of legislation and business leadership*,* who told them that it would cost them more [to deal with it properly]. So then it was nice that he could say: but this is company policy.* (Interview C)	*Inelastic targets* *Non-divisibility*

**Table 6:** California effect

Finding	Illustrative interview quotes	Five elements of Bradford [3]
If a US company is driven by legislation, it is often from California.	*“…sometimes we get The State of California has some pretty strict guidelines around what they can and can’t use*,* so we will have to sometimes provide them with like*,* oh*,* this will last for this long*,* and this is nontoxic. […]* (Interview B). *“I worked at 3 different retailers*,* similar but different*,* and I think all companies are the same. We’re willing to meet the more stringent law and apply it across all of our retail spaces. So when California rolls out a law*,* it’s typically easier for us to meet California and then apply it out everywhere else…l.”* (Interview K) *“. in the States it may have to come from regulation*,* and it will probably come from states like California and New York*,* and those types of more blue States were sort of… I think California is an obvious one.”* (Interview J)	*Market size* *Regulatory capacity* *Stringent standards* *Non-divisibility*
Regulation forces adoption of sustainable practices, which drives new vendor relationships	*“But if you go to certain parts of the country*,* they may have more strict state regulations which makes it easier to be you know*,* basically forced into adoption of sustainable practices like on the West Coast*,* for instance*,* there’s quite a bit more regulation in California than there is.you know*,* even in Minnesota*,* for example. So it’s actually really challenging from a business operation perspective [in the US]*,* because you have to look at so many different vendors to see if you can patchwork a solution together.”* (Interview P)	*Stringent standards* *Inelastic targets* *Non-divisibility*
California as a reflection of European policy	*“I saw in the EU*,* where with the vacuum cleaners energy requirements* […] *it was no longer a power race*,* put more power in*,* so that you had to design the system to be more efficient* […] *regulation has been a big driving force*,* I think*,* like the right to repair. And those sort of things. I think they start in Europe*,* and they will make their way to the US. So I think definitely legislation*,* I think. See California as as sort of a mirror of Europe a little bit in that respect* (Interview C)	*Market size* *Regulatory capacity* *Stringent standards* *Inelastic targets* *Non-divisibility*

### Barriers and Strategies to Overcome them

Interviewees routinely reflected on legislative barriers to the successful implementation and scaling of circular business models in the United States (Table 3). The lack of a comprehensive U.S. policy framework (particularly as compared to Europe) was a recurring theme. Interviewees often mentioned they were ‘following Europe’: *“We’re usually behind Europe a little bit. So it’s just something to keep my eye on*” (interview A). Sometimes interviewees struggled to make their business models work in the U.S. regulatory environment, where they considered incentives and legislation to be comparatively unfavorable for circular business models as compared to the European context.

At the same time, the interviews revealed a number of ways in which companies are working to overcome these challenges through various forms of institutional work [63], including driving legal support for their business models, developing their own rules and regulations, working to leverage and support emergent legislation, and collaborating with key stakeholders. This has included discussion with regulators on how laws should be interpreted, and networking strategies with government, NGOs and academia to move forward. Corporate efforts have included both strategies to adopt legislation or finding alternatives to legislation (see Table 4).

### The Brussels and California Effects

We observed the Brussels effect influencing companies’ operations in the United States (Table 5), as well as broad awareness of the California effect (Table 6), with a number of interviewees referring to emergent legislation in California as driving adoption of circular practices (e.g. with vendors and industry partners). Regarding EU policies, several interviewees stressed that U.S. policy will follow the EU, or that compliance with EU laws is needed in order to do business in Europe. There were also examples when companies with headquarters in Europe influenced practices in the U.S. branch, like in Company C where its senior leadership drove CE innovation from Europe to other country contexts like the US as “company policy” (Table 5).

Regarding the California effect, comments from interviewees revealed that California is often at the forefront in setting policies in the U.S. Some interviewees also noted how some Californian policies “mirrored” those of the EU, a tendency that has also been noted in news media [42].

### The Special Case of EV Battery Recycling

A notable exception to the lack of circular economy policy in the United States was the Inflation Reduction Act (IRA) and its role in incentivizing EV battery recycling. As noted by interviewees:

*[Regarding battery recycling] “I think what we definitely saw with the IRA was that Europe was really at the front*,* run front runner on the investment point of view. Then when the IRA came in*,* they started to say*,* oh*,* OK*,* actually*,* let’s jump to us and then Europe was kind of pushed from a CapEx investment point of view (…) with partners of big CapEx investments*,* they’ve said actually now we’ve shifted priority. We’re first moving into the US and then we’ll move into Europe. So definitely I already had a big impact on that*,* that’s for sure.” (Interview M)*.

*”I mean. there’s gonna be a tremendous number of battery regulations that are coming. And you know our ask of the US Congress and Senate is that if any battery legislation come up*,* that they contact ISRI. You know*,* to help us craft that legislation*,* because you know*,* they have members that process that material“(interview H).*

The IRA was thus perceived by interviewees as driving circular economy innovation, although the environmental component of the legislation was primarily aimed at supporting climate and energy objectives rather than circular economy.

### The Role of Consumers in the United States

Finally, interviewees referred to the difference between EU and U.S. customer personas, with one even suggesting the “American dream” was somewhat incompatible with certain types of circular consumer offerings (e.g. product-as-a-service models):

*“We found that American consumers make decisions probably different than European consumers. Do you know*,* I think there’s what I’ll call a plague of fast fashion [in the USA].”* (Interview O).

*“The American dream and rental…[.] It’s still largely looked down upon because I think of the rental center atmosphere. But for millennials and Gen. Z. You know*,* they’re very keen on it. It’s just getting the word out.”* (Interview D).

On the other hand, circular business-to-business practices like remanufacturing are much more embedded in business practices in the United States:

*“Part of it is because we have been doing it for so long in the United States…. We’ve really proven the model there. And again*,* our customers in the US very much trust the product. They’ve seen the quality that we’ve provided and they’ve seen*,* you know the the cost savings that they’re able to get by buying this remanufactured product and how they’re helping the economy.”* (interview G).

It is therefore an open question to what extent differing consumer preferences in the United States. might create barriers for the implementation of business-to-consumer (B2C) circular business models.

## Discussion

### Contributions to CE Policy

Many circular business models need support from policies, or at the very least that current laws, policies, and standards are not posing barriers for developing circular offerings [16]. The EU has developed a more elaborate policy framework for the CE than the United States, and this was also obvious in the interviews, where several interviewees stressed the need for the United States to adopt similar policies. But interviewees also mentioned ways to address current barriers in the United States, including dialogues with policymakers on the interpretation of laws, and collaborations to move things forward. Lobbying, developing own rules and collaboration with relevant actors were key examples to pursue circular business models in sometimes adverse policy contexts.

Yet, in Europe, state-level environmental legislation often outpaces European legislation [2]. In the EU, countries like France are “policy innovators”, testing national policies that are often later applied at the EU level, often with the intention to harmonize national practices in the EU. Our analysis revealed that similar mechanisms apply in the United States, where pioneer states like California adopt progressive practices and others follow. We observe a clear ‘California effect’, in the sense that companies operating U.S.-wide need a license to operate in the most stringent environmental contexts (in this case, California) and this affects their innovations across the whole company. However, many laws are still under the jurisdiction of the states, and thus we do not see the same drive towards the harmonization of practices in the United States as is the case in the EU. The fact that the EU Internal Market — and the free trade of goods — is considered as the perhaps greatest success of the EU, can explain the strong harmonization efforts in the EU.

### Contributions to CE Innovation in a Business Context

We found that besides the importance of policy, the effect of globalization and the power of internationally operating companies should not be underestimated. Organizations with a head office in Europe or Asia were found to drive CE innovations in the United States through company policy (Table 5). This was not only the case for multinationals but also smaller internationally operating businesses who drove change through their policies.

Importantly, when introducing circular business models in the United States, we also found that companies often encountered a different type of customer, in particular in the B2C context, where customers are used to ‘fast consumption’ and product ownership that does not fit many European circular innovations. Circular scale-ups with an origin in the United States (e.g. companies I & O) identified new markets with younger generations whose lives are more suited for new business models such as furniture rental. This echoes earlier research on the importance of the socio-cultural context in which circular business models are introduced [65], as well as more broadly institutional theory and work to make innovations fit in new contexts [62, 66]. It also goes back to traditional marketing practices such as market segmentation and identifying new and emerging market segments for new circular propositions. See for instance the work by Mugge et al. (2017) who identified different customer segments for refurbished smartphones [67], Magnier et al. (2019) who identified different customer clusters for products made from ocean plastics [68], and Zink & Geyer (2017) who explored the substitution effects of circular products to avoid rebounds [69]. This implies that companies might influence CE practices in the U.S. through a foreign head office, but traditional marketing tactics need to be applied to make the CE innovations successful in other contexts.

### Contributions to Theory: Reflecting on the Brussels and California Effects

In this study, we found evidence for the Brussels as well as California effects. Connecting this to the five elements of Bradford [3], an important effect seems to be related to non-divisibility: the fact that internationally operating businesses cannot ‘pick and choose’ and rather make their products and services market-ready for the most stringent regulatory frameworks. Bradford posits this as a necessary condition for the Brussels effect, triggered when the benefits of designing for adherence to the most stringent standards outweigh the associated costs. This appeared to be the case for the large companies in our sample. The European market size coupled with the regulatory capacity and stringent targets seem to influence the ability of ‘Brussels’ to exercise influence on circular innovations in the USA.

Likewise, companies follow legislation in California (and to a lesser extent New York State), whose policy is seen as a precursor for more stringent environmental policy elsewhere in the USA. And as a rather large market, it seems to be difficult to avoid California and therefore its legislation, again pointing at the non-divisibility effect. Linked to this, we found evidence in the interviews for the ‘de facto’ Brussels Effect because European policies or practices by the companies’ European facilities influenced their practices in the USA. The de facto Brussels effect **(**Bradford [3]) happens when companies operating outside the EU voluntarily adopt practices that adhere to EU rules (e.g. starting to innovate for the circular economy), as they want to be sure to be able to sell their products in the EU.

Finally, as part of desk research in preparing the landscape table of CE policy elements in Europe, the U.S. and California (Table 1), we observed what Bradford [3] refers to as a ‘de jure’ Brussels effect, whereby EU policy influences the development of similar policy in other jurisdictions. This appears to be the case for California SB-707, the first Extended Producer Responsibility legislation of its kind in the U.S. for textiles. While individual member countries throughout the EU have introduced EPRs in the past (e.g. France, Netherlands), the EU introduced a harmonized proposal for textile EPR in 2023 as part of the Strategy for Circular and Sustainable Textiles. California’s SB-707 shares many similarities with the latter related to funding mechanisms, producer responsibility, infrastructure investment, and stringency. This observation was further supported by interviewees’ reflections, for example that California is “a mirror of Europe” when it comes to environmental policy.

### Limitations and Future Research

A wave of technological and organizational innovation for the circular economy is underway in the United States. In addition to EV battery recycling, companies are working on compostable plastic alternatives, AI for recycling sorting, reuse and remanufacturing technology, wastewater treatment, and broader corporate strategy around circularity [70]. While circularity outcomes might appear straightforward and easy to measure (e.g., recycling battery materials to reduce the need for new critical minerals in the supply chain), experience dictates that positive circularity outcomes are not always guaranteed. For example, CE innovations are often not assessed for quantitative outcomes by companies [71] or academia [72]. Unintended rebound effects may occur e.g., due to products not being suitable substitutes for linear alternatives [69, 73], the availability of recycled materials, and the energy needed for circular solutions [74], which may or may not be fossil-fuel based [72].

While the present work was exploratory and consisted of a heterogeneous sample set of companies to get a broad view, it confirmed the existence of the Brussels and California effects. Future work could investigate these effects through longitudinal analysis and take stock of the number of circular innovations and impact of these innovations. For example, to what extent did they replace linear business models, or do circular business models mainly co-exist with dominant linear business models [75]? What is the customer adoption of novel circular business models in the United States? What type of customer segmentation is needed to address real customer needs with desirable, but also viable, feasible and circular business models [76]? How can specific CE policies in the United States more directly phase out dominant linear models? Finally, future studies could use life cycle assessment as part of a mixed methods toolkit to measure the total impact of these technologies, both at the firm level and at the broader ecosystem level. This type of data will be important for helping to shape effective policies and incentives that drive net environmental improvements. Recent advancements in leveraging machine learning for environmental impact assessment can help to speed up this work and increase its scale [77].

### Suggestions for Policy & Business

The study confirmed earlier findings from literature that current policies and laws, and how laws are interpreted, can act as barriers for circular business offerings. Policymakers thus need to consider how to support CE solutions. As it may be unrealistic to expect all jurisdictions to have similar policies, it could be useful to have policies in jurisdictions with a large market - like the EU and California - establishing the blueprint for other actors. To the extent that these policies are well designed, they may then have welfare-enhancing effects [3]. The literature, and in some cases also the interviewees, indicate that California is also heavily influenced by EU policies, and more consistency between the policies leads to a better business environment. Businesses should therefore lobby for increased consistency of policies and laws in different jurisdictions.

For policy makers we suggest that if there is a deliberate choice to adopt a certain policy in another context, caution should be taken in terms of cost and context [50]. There is always a trade-off between cost and impact, and the right policy mix also “varies with socio-economic, cultural and political characteristics of the society” [50, 10]. Therefore, a more harmonized legal framework in the EU is good for trade, as is coordination of laws and policies adopted in the EU and the US.

In the US, both the CHIPS and Science Act and the White House Office of Science and Technology Policy (OSTP) Net-Zero Game Changers Initiative have acknowledged the essential role that the circular economy will play in delivering on U.S. net zero goals [73]. A 2023 White House roundtable between the OSTP and Ellen MacArthur foundation emphasized the roles of technological innovation, systems innovation and experimentation, and new business models and policies in transitioning to a circular economy [44, 78]. While this is encouraging to see, it is still unclear what concrete policies will emerge. So far, policies incentivizing EV battery recycling seem to be driving rapid circularity improvements in this sector in the United States. There may be lessons here for policymakers to encourage similar progress in other U.S. sectors and industries where circularity is important from both a climate and security perspective. In several important policy areas related to the CE - such as producer responsibilities for recycling, and right-to-repair policies - it is the States in the US that adopts a majority of the rules, and the US policy mix is less advanced than in the EU (for the case of right-to-repair policies, see [36]). The US could then benefit from a more coordinated approach, driven more at the federal level, though such policy processes may be compromised due to political conflicts.

## Conclusions

We investigated the following question: To what extent are companies operating in the United States influenced by circular economy policies outside their direct context? In this study we found evidence for positive environmental and circular policy spillovers, dubbed the Brussels and California effects. We found lack of a comprehensive policy framework, opposition by other business and adverse policy contexts to be the key barriers to circular innovation in the United States. Collaboration, developing own standards, and lobbying were seen as key industry solutions. The study also reinforced the need to understand the socio-cultural context to develop desirable, but also feasible, viable and circular solutions. We conducted a first exploratory study with diverse examples. Future work could build on this exploratory work to identify the impacts of policy spillovers more deeply. Moreover, future research can contribute to the development of more comprehensive environmental and circular policy frameworks in the United States to stimulate the CE transition.

## Data Availability

Not applicable.

## Abbreviations

B2C: business-to-consumer
CBMs: Circular Business Models
CE: Circular Economy
CSRD: Corporate Sustainability Reporting Directive
EU: European Union
EV: Electric Vehicle
IRA: Inflation Reduction Act
OE: Original Equipment
OEM: Original Equipment Manufacturer
PACE: Platform for Accelerating the Circular Economy
SMEs: Small & Medium Sized Enterprises
U.S.: United States
